# *PRKN*-linked familial Parkinson’s disease: cellular and molecular mechanisms of disease-linked variants

**DOI:** 10.1007/s00018-024-05262-8

**Published:** 2024-05-20

**Authors:** Lene Clausen, Justyna Okarmus, Vasileios Voutsinos, Morten Meyer, Kresten Lindorff-Larsen, Rasmus Hartmann-Petersen

**Affiliations:** 1 Department of Biology, Linderstrøm-Lang Centre for Protein Science, University of Copenhagen, 2200 Copenhagen, Denmark; 2 Department of Neurobiology Research, Institute of Molecular Medicine, University of Southern Denmark, 5230 Odense, Denmark; 3 Department of Neurology, Odense University Hospital, 5000 Odense, Denmark; 4 Department of Clinical Research, BRIDGE, Brain Research Inter Disciplinary Guided Excellence, University of Southern Denmark, 5230 Odense, Denmark

**Keywords:** MAVE, DMS, Protein folding, Protein stability, Protein degradation, Protein quality control, Proteasome, Ubiquitin, Parkinson’s disease, AR-JP, PARK2, PRKN, VUS, Mitochondria

## Abstract

Parkinson’s disease (PD) is a common and incurable neurodegenerative disorder that arises from the loss of dopaminergic neurons in the *substantia nigra* and is mainly characterized by progressive loss of motor function. Monogenic familial PD is associated with highly penetrant variants in specific genes, notably the *PRKN* gene, where homozygous or compound heterozygous loss-of-function variants predominate. *PRKN* encodes Parkin, an E3 ubiquitin-protein ligase important for protein ubiquitination and mitophagy of damaged mitochondria. Accordingly, Parkin plays a central role in mitochondrial quality control but is itself also subject to a strict protein quality control system that rapidly eliminates certain disease-linked Parkin variants. Here, we summarize the cellular and molecular functions of Parkin, highlighting the various mechanisms by which *PRKN* gene variants result in loss-of-function. We emphasize the importance of high-throughput assays and computational tools for the clinical classification of *PRKN* gene variants and how detailed insights into the pathogenic mechanisms of *PRKN* gene variants may impact the development of personalized therapeutics.

## Introduction

Parkinson’s disease (PD) is the most common movement disorder and the second most common neurodegenerative disorder after Alzheimer’s disease [1,2], affecting 2–3% of the population aged ≥ 65 years [1]. The etiology of PD is still not clear, although research suggests that the cause is multifactorial and linked to environmental agents, gene variants and aging [1].

Pathologically, PD is the result of the progressive loss of ventral mesencephalic dopaminergic neurons, which causes striatal dopamine deficiency and impairment of motor control [3]. Accordingly, there is an estimated 30% loss of midbrain dopaminergic neurons at the onset of motor symptoms [4]. The primary motor symptoms include bradykinesia, rigidity, tremor and at later stages postural instability [3]. In addition, PD patients also display a broad spectrum of non-motor features that typically develop gradually for years before any motor symptoms appear, including autonomic dysfunction (e.g. constipation), loss of the sense of smell and sleep impairment [5].

Currently, efficient therapies are available, making PD the first neurodegenerative disorder to be successfully managed and increasing the quality of life for patients for many years after disease onset [1]. However, the treatments are not curative and as a result the disease will slowly progress and eventually cause severe disabilities. The main therapeutic approaches include dopamine-related pharmacological treatment for PD motor symptoms, the most common being Levodopa (l-DOPA) [1,5]. However, l-DOPA treatment is complicated by l-DOPA-induced dyskinesia, motor fluctuations, and l-DOPA-resistant motor function [1]. For all PD patients the effect of medication wears off over time, hence only alleviating the symptoms for a limited number of years after diagnosis.

Often, the affected neurons in PD accumulate misfolded and aggregated α-synuclein into intracellular inclusions termed Lewy bodies, which appear to contribute to PD pathogenesis. Thus, both degeneration of dopaminergic neurons and the presence of Lewy bodies, constitute the hallmarks of PD [5]. However, since Lewy bodies are also present in other diseases such as dementia with Lewy bodies (DLB) [6] and are rarely seen in certain familial subtypes of PD [7], their formation does not solely account for the neuronal cell loss observed in PD. Another important mechanism contributing to PD, as well as other neurodegenerative diseases such as Huntington’s disease and amyotrophic lateral sclerosis, is mitochondrial dysfunction [8,9]. The initial evidence of altered mitochondrial function associated with PD emerged when mitochondrial toxins were observed to induce acute PD-like symptoms [10]. Later, this link was supported by the identification of genes associated with monogenic forms of PD. Here, the associated genes were found to encode proteins involved in mitochondrial quality control and the degradation of damaged mitochondria [11], supporting mitochondrial dysfunction to be implicated in the pathology of PD and a likely mechanism contributing to the neuronal cell loss [12].

Although a large majority of diagnosed PD cases are idiopathic, autosomal dominant and recessive familial forms have been identified [13]. Common genetic variability at more than 90 loci has been linked to PD, accounting for 16–36% of the heritable component of the disease. However, individually, each of these loci only has a small effect size. Conversely, rare, but highly penetrant, genetic alterations in *SNCA (PARK1/4)*, *LRRK2 (PARK8)*, *VPS35 (PARK17)*, *DJ-1 (PARK7)*, *PINK1 (PARK6)* and *PRKN (PARK2)* have been linked to monogenic familial PD [13,14]. Obviously, the identification of disease-linked germline variants in these genes is paramount for accurate diagnosis and genetic counselling of affected individuals and their families. Moreover, characterizing the mechanisms by which disease-linked variants function will enhance our understanding of PD in general and potentially contribute to the development of future therapeutic strategies. In this review, we start by providing a brief introduction to *PRKN-*linked familial PD and the relevant molecular and cellular pathways, noting that these topics have been explored in several excellent in-depth and historical reviews [12,15–20]. Subsequently, we delve into the molecular mechanisms underlying *PRKN* loss of function and recent advances in computational prediction tools and high-throughput technologies for clinical assessment of *PRKN* gene variants.

## *PRKN*-linked Parkinson’s disease

*PRKN* (also known as *PARK2*) is a well-characterized PD-linked gene, encoding the E3 ubiquitin-protein ligase Parkin [21,22] involved in mitochondrial quality control and the degradation of damaged mitochondria through mitophagy. *PRKN* gene variants associated with PD lead to a loss of Parkin function and range from single base pair substitutions to small deletions and splice site aberrations, to deletions that span thousands of nucleotides [23]. Obviously, gene variants with altered splicing or deletions of coding regions are expected to disrupt gene function completely or at least very strongly, and similarly strong effects are expected for single nucleotide substitutions resulting in early stop codons (nonsense variants). Conversely, the effects of missense variants, where one amino acid residue is exchanged with another, may range from a complete loss of function to neutral (harmless), and intriguingly even to enhanced activity [24,25].

*PRKN* variants are the most common cause of autosomal recessive PD (ARPD) regardless of gender and with an age of onset before 40 years of age in most individuals [21,26,27]. In addition, *PRKN* variants are the most common cause of early-onset PD (age of onset ≤ 20 years) [26] accounting for 42% of the familial early-onset PD cases [27] and the majority of *PRKN-*related PD patients with disease onset before 20 years of age carry bi-allelic *PRKN* variants [28]. In terms of the cardinal motor symptoms, *PRKN*-related PD patients demonstrate a clinical phenotype resembling that of sporadic PD patients [29]. The *PRKN*-specific clinical features include early disease onset, hyperreflexia and dystonia as some of the first signs of the disease besides slow disease progression [29]. Additionally, dementia is rarely reported [30]. Clinical pathology shows a selective loss of dopaminergic neurons in the *substantia nigra* and loss of noradrenergic neurons in the *locus coeruleus* with accompanying gliosis [31] without the presence of Lewy bodies, except in rare cases [7]. In most cases, *PRKN*-related PD patients respond well to l-DOPA treatment even at late stages, and L-DOPA is therefore often considered an efficient treatment for a sustained period of time. *PRKN*-linked PD patients in treatment with L-DOPA are, however, prone to develop l-DOPA-induced dyskinesia [26,30].

Induced pluripotent stem cells (iPSCs), derived from somatic cells or tissues, have enabled the investigation of the progressive development of disease-associated cellular changes in dopaminergic neurons from PD patients. Concurrently, advances in genome editing techniques have allowed correction or introduction of disease-causing variants in PD-relevant genes of importance for mitochondrial function. This in turn enables examination of isolated effects of PD-relevant protein variants, including Parkin, in developing and mature human dopaminergic neurons. Studies of PD patient iPSC-derived neurons with *PRKN* variants have documented increased oxidative stress, abnormal mitochondrial/lysosomal morphology and function, dysregulation of dopamine homeostasis, and to some extent α-synuclein accumulation [32–38].

## Parkin structure and function

**Fig. 1:**
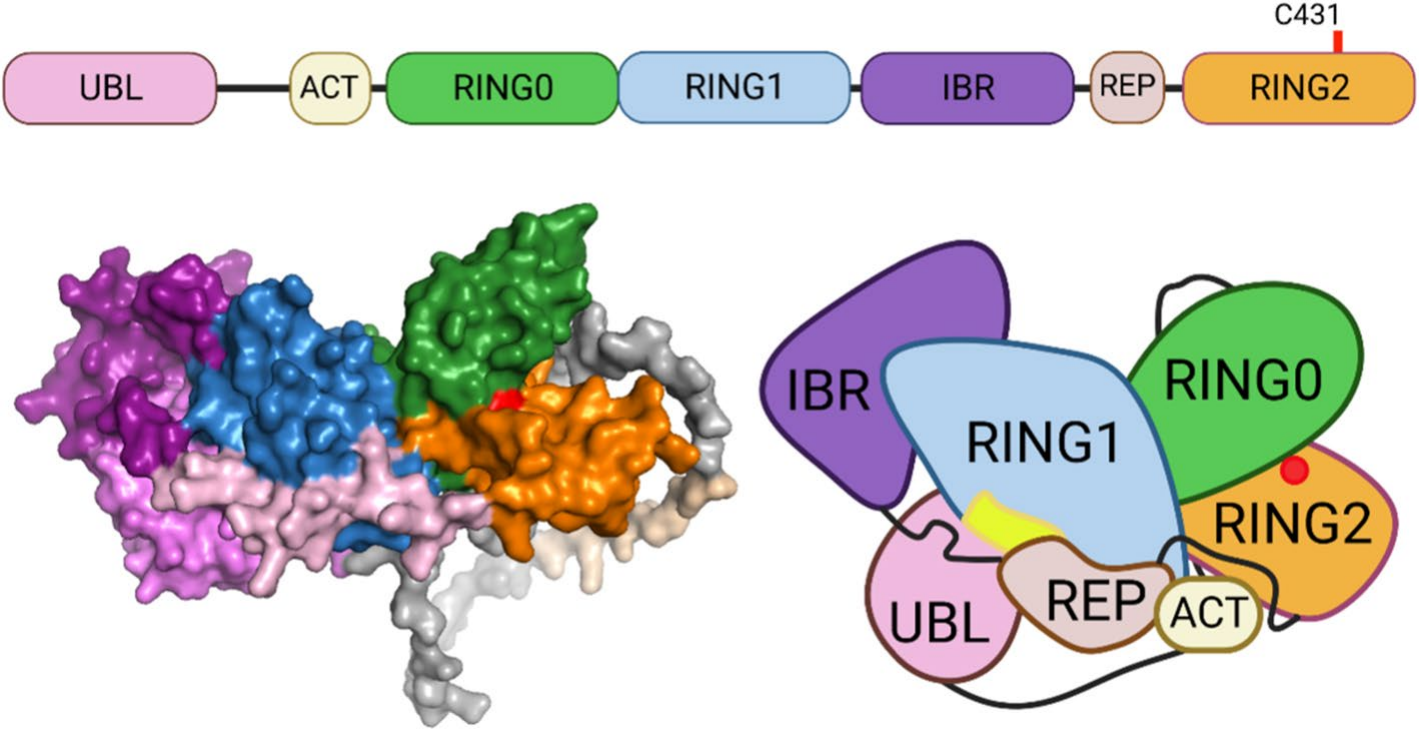
Parkin domain organization and auto-inhibited conformation. Schematic representation of the Parkin domain organization (upper panel). The AlphaFold2 (AF-O60260-F1) structure of Parkin is depicted, with colors corresponding to the domain organization (lower-left). Simplified representation of the Parkin structure in its auto-inhibited conformation (lower-right). In this conformation, both the active site (red dot within RING2) and the E2 binding site (yellow region in RING1) are occluded, thus preventing Parkin activity. Figure created with BioRender.com

Parkin is a soluble multi-domain protein comprised of 465 amino acid residues [22], which make up an N-terminal ubiquitin-like (UBL) domain and four RING-like domains; RING0, RING1, in-between RING (IBR) and RING2 (Fig. 1). This arrangement of RING-IBR-RING (RBR) domains categorizes Parkin within the RBR E3 ubiquitin ligase protein family. Each RING domain co-ordinates two zinc ions that are critical for maintaining the protein structure [39]. In addition, Parkin contains a conserved nine-residue motif named the activating element (ACT) (residue 101–109) in the linker region between the UBL and RING0 domains [40], as well as a so-called repressor element (REP) located just upstream of the RING2 domain [41] (Fig. 1). These Parkin domains and motifs are highly conserved across species and play key roles for activity and regulation [40].

As an E3 ubiquitin ligase, Parkin’s enzymatic activity is to catalyze covalent conjugation of the small protein ubiquitin to various cellular proteins. In this process, termed ubiquitination [42–45], a ubiquitin-loaded E2 enzyme will, in collaboration with an E3 enzyme, catalyze conjugation of the ubiquitin to the target protein. Typically this conjugation occurs via an isopeptide bond between the C-terminal carboxyl group of ubiquitin and the amino group of a lysine side chain in the target protein. Some E3 families catalyze the transfer of the ubiquitin moiety directly from the E2 to the target protein, whereas for others, including RBR-type E3s, such as Parkin, ubiquitin is first bound via a thioester bond to a cysteine residue in the E3, and subsequently transferred to the target protein [46–48]. In the case of Parkin, the active site cysteine residue is C431 located in the RING2 domain, while E2 binding occurs to the RING1 domain. Additional rounds of ubiquitination will target one or several of the lysine residues (K6, K11, K27, K29, K33, K48 and K63) in the conjugated ubiquitin, and lead to the formation of ubiquitin chains. Both cellular and in vitro studies have shown that Parkin catalyzes the formation of K6-, K11-, K48- and K63-linked ubiquitin chains [18]. Protein targets of Parkin catalyzed ubiquitination include several outer mitochondrial membrane (OMM) proteins including mitofusins MFN1 and MFN2 [49–51] but also a range of cytosolic proteins. This ubiquitination marks them for proteasomal degradation and autophagy, eventually leading to the clearance of damaged mitochondria (see below).

Considering these important cellular functions of Parkin, it is not surprising that its activity is tightly regulated and requires phosphorylation and structural rearrangements to become active [18,20,52]. Under basal conditions, Parkin adopts a closed, auto-inhibited conformation facilitated by its UBL domain associated with RING1. This auto-inhibitory state is governed by two key interactions: the REP element and UBL domain occluding the E2 binding site in RING1 and the RING0 domain blocking the active site (C431) in RING2 [53,54] (Fig. 1).

**Fig. 2:**
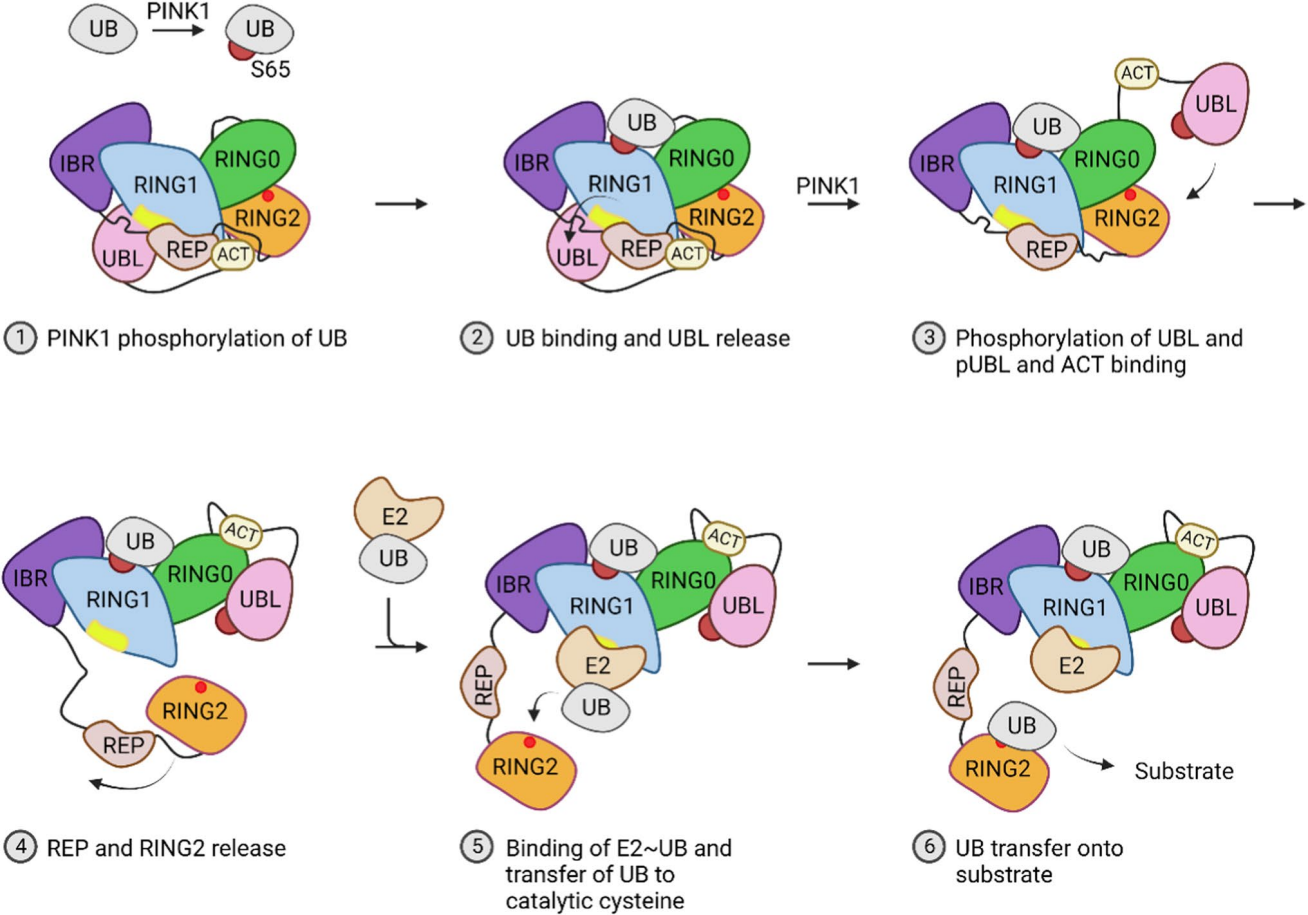
Parkin activation. Parkin in its auto-inhibited conformation has the active site (red dot within RING2) and the E2 binding site (yellow region within RING1) occluded. PINK1 phosphorylation (red) of ubiquitin (UB) and the subsequent binding of phosphorylated ubiquitin result in the release of the UBL domain, priming it for phosphorylation by PINK1. Once phosphorylated, the UBL and ACT elements bind to RING0, leading to the release of REP and RING2. This release allows for the binding of E2 ~ UB, facilitating the transfer of ubiquitin from E2 to the catalytic C431 residue (red), and finally, the transfer of ubiquitin onto the substrate. Figure created with BioRender.com

The structure of the individual domains and full-length Parkin have been solved [40,41,55–62], and the crystal structure of Parkin in its autoinhibited state is highly similar to the structure predicted by AlphaFold. Upon Parkin activation, auto-inhibited Parkin binds to ubiquitin phosphorylated by PTEN-induced kinase PINK1 (encoded by *PINK1*, the second most commonly ARPD-linked gene) at position S65 and translocates to damaged mitochondria. Parkin binding to phosphorylated ubiquitin retains it in an auto-inhibited conformation [55,58] but induces structural rearrangements of the UBL domain. In turn, this structural rearrangement allows for efficient Parkin phosphorylation by PINK1 at S65 in the UBL domain (the equivalent position in the UBL domain to S65 in ubiquitin) [41,55,57–59,63–66]. Next, binding of the phosphorylated UBL domain to a phospho-binding pocket in RING0 and the binding of the ACT element (previously bound by RING2 and REP) to RING0 cause a major structural reorganization that releases RING2 and the catalytic C431 residue from its inhibited state. This transforms Parkin into the fully activated Parkin conformation and promotes the binding of a ubiquitin-loaded E2 [24,40,54,57,60]. Subsequently, activated Parkin will ubiquitinate numerous OMM proteins [49–51] (Fig. 2).

## Parkin regulates mitochondrial dynamics

Maintaining cellular homeostasis in response to stress conditions is crucial for cell function and survival. Mitochondria are double-membrane enclosed compartments that constantly adapt their shape and function to support the cell’s demands [67,68]. Since mitochondria generate high levels of reactive oxygen species as a natural by-product to their energy production, this makes them highly susceptible to mtDNA mutations and protein misfolding. Consequently, efficient and accurate quality control mechanisms are crucial for maintaining a healthy mitochondrial network and homeostasis [69]. This is of particular importance for non-dividing cells, like neurons, that are unable to dilute faulty mitochondria through cell division. These mechanisms include activation of the mitochondrial unfolded protein response (mtUPR) and integrated stress response (mtISR) pathways, as well as retro-translocation of misfolded mitochondrial proteins followed by degradation via the ubiquitin–proteasome system (UPS) (reviewed in [70–72]). These mechanisms provide an early first response system to alleviate the mitochondrial stress situation. However, as an additional layer of defense, cells also tightly regulate the biogenesis and degradation of mitochondria. Since mitochondria cannot be synthesized de novo, damaged or non-functional mitochondria are replaced by pre-existing ones, involving both fusion and fission processes. The balance between fusion and fission events affects the number, size and morphology of individual mitochondria, including small spherical shapes, long or short tube-like structures, or connected tubules [73,74]. Occurring on a regular basis, the two opposing processes, fusion and fission, comprise the mitochondrial dynamics.

Mitochondrial membrane fusion is orchestrated by three proteins belonging to the dynamin superfamily. The outer mitochondrial membrane (OMM) mitofusins, MFN1 and MFN2, required for fusion of the OMM, while the inner mitochondrial membrane (IMM) protein optic atrophy-1, OPA1, is responsible for fusion of the IMM. Fission occurs in response to cell division and stress conditions and requires the translocation of the dynamin-related protein 1, DRP1, to the OMM, where it divides the mitochondria into two [9,75]. Two distinct fusion events have been described: complete fusion and partial fusion [76]. Partial fusion and fission events ensure a continuous exchange of content between mitochondria, ensuring a homogenous mitochondrial population within the cell [9]. Although the function in mitochondrial quality control needs further exploration, based on the current understanding, fusion and fission events are assumed to aid the separation of damaged mitochondrial components from the functional mitochondrial network [74]. A recent study has demonstrated how the mitochondrion may display different membrane potentials along the mitochondrion [77,78], supporting the possibility of a mitochondrion having a high membrane potential in one part and a low membrane potential in another. This could explain the consistent observation of uneven fission events [79]. Here, daughter units displaying a high membrane potential were likely to reintegrate into the mitochondrial network by fusion, whereas daughter units exhibiting low membrane potentials were isolated from the network and subsequent likely to be eliminated by a selective form of autophagy, termed mitophagy.

**Fig. 3:**
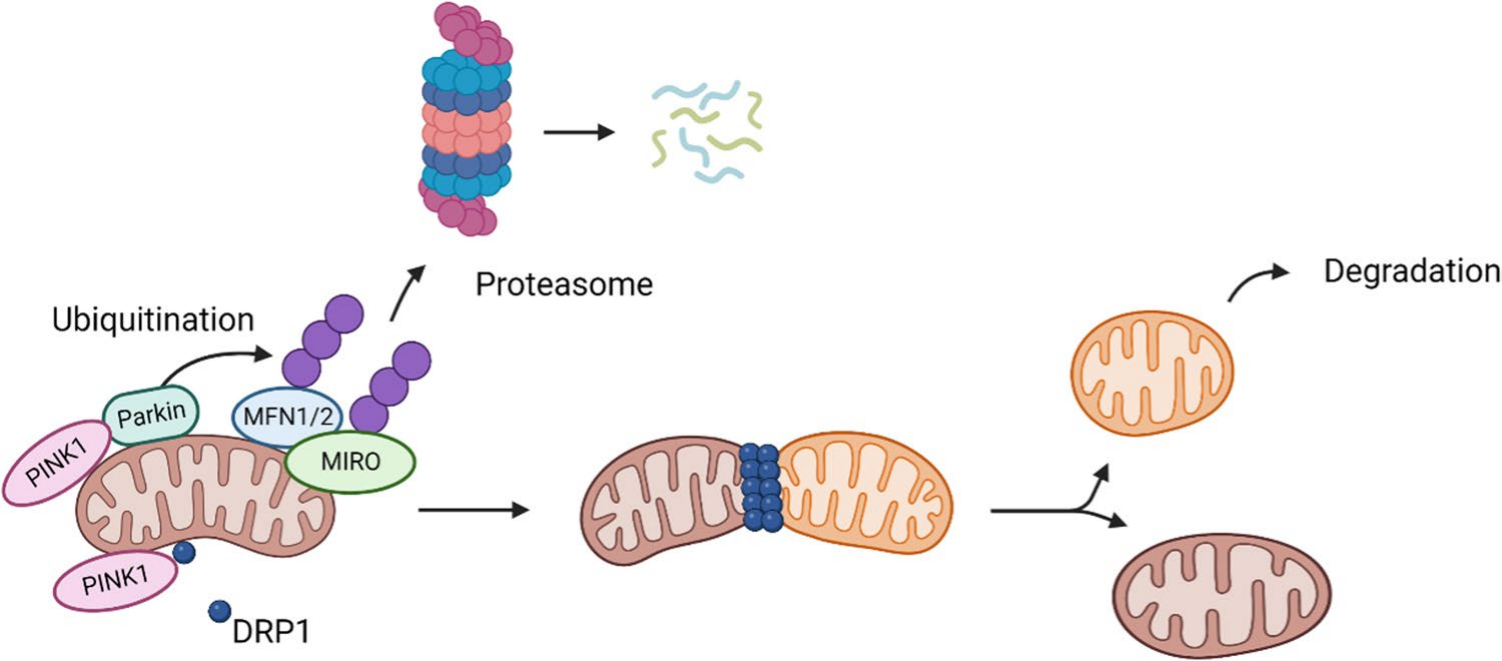
The role of Parkin in mitochondrial fission. Mitochondrial fission and subsequent degradation of damaged mitochondria (orange mitochondria) by Parkin/PINK1 dependent attachment of ubiquitin (purple) and consequent proteasomal degradation of mitofusins (MFN1/2) and MIRO, and PINK1 mediated recruitment of DRP1. Figure created with BioRender.com

Mitophagy is the process of autophagy-mediated selective degradation of abnormal or dysfunctional mitochondria in lysosomes, is a critical pathway for sustaining a network of healthy mitochondria. Parkin and PINK1 play pivotal roles in preventing fusion and promoting the segregation of damaged mitochondria for degradation through mitophagy [51,80–83]. To this end, Parkin mediates the ubiquitination and subsequent proteasomal degradation of multiple OMM proteins, such as MIRO, MFN1 and MFN2, important for mitochondrial fusion. At the same time, PINK1 induces mitochondrial fission by recruiting DRP1 to the OMM [84–86] (Fig. 3).

Overall, Parkin can preserve mitochondrial integrity by regulating mitochondrial dynamics by mediating mitochondrial fission [51,80–83,87]. Mitochondrial fission is widely recognized as the initial step necessary for removal of damaged mitochondria via mitophagy [84,88].

## PINK1 and Parkin-mediated mitophagy

Work in *Drosophila* provided the first links between PINK1, Parkin and mitophagy [89–91], showing that *parkin* and *pink1* null mutants display similar phenotypes, including locomotor defects and male sterility, and linking these phenotypes to defects in mitochondrial morphology [89,90]. Later proteomics studies revealed that mitochondrial protein turnover was slowed in *parkin* mutants [72], thus showing that Parkin promotes mitophagy in vivo.

Accordingly, Parkin and PINK1 not only promote the segregation of damaged mitochondria, but also maintain mitochondrial homeostasis by mediating the degradation of damaged mitochondria [92,93]. When mitochondria are healthy, PINK1 is targeted to the mitochondria through its mitochondrial targeting sequence (MTS) [92]. Subsequently, it translocates through the translocase of the outer membrane (TOM) complex into the translocase of the inner membrane (TIM) complex [94]. Here, PINK1 is cleaved by the mitochondrial processing protease (MPP) [95], which catalyzes the cleavage of the N-terminal MTS of imported precursor proteins [96]. Following this, PINK1 is rapidly cleaved in its N-terminal transmembrane domain by the mitochondrial intramembrane PARL protease [97,98] and is retrotranslocated from the mitochondria to the cytosol for degradation through the ubiquitin–proteasome system (UPS) via the so-called N-end rule ubiquitin-conjugation pathway [99]. This PINK1 degradation mechanism ensures its low abundance in cells containing healthy mitochondria.

During conditions where mitochondria become damaged or dysfunctional, it is critical that these organelles are cleared from the cell. Defects in mitochondrial protein import may occur in both depolarized mitochondria (e.g., due to mitochondrial damage) [100] and polarized mitochondria (e.g., defective due to perturbed mitochondrial proteostasis) [88,101–103]. Impairment of protein import into the mitochondria prevents the translocation of PINK1, leading to PINK1 accumulation on the OMM in association with the TOM complex [104], thus flagging these mitochondria as damaged. Then, PINK1 on the OMM is activated through dimerization and trans-auto-phosphorylation [105–107]. Recent structural studies of PINK1 in an activated state have provided detailed insights into the order of events during PINK1 activation [107,108].

A recent study indicates an early first step in Parkin-PINK1 dependent mitophagy, prior to PINK1 activation of Parkin, involves the AMP-activated protein kinase AMPK that regulates autophagy and mitophagy through the serine/threonine protein kinase, ULK1 [109]. Here, phosphorylation of the highly conserved S108 in the Parkin ACT element by ULK1 appears to precede PINK1 activation of Parkin in response to mitochondrial damage [110]. Subsequently, PINK1 triggers Parkin activation a multi-step process. First, PINK1 phosphorylates S65 on pre-existing ubiquitin on the OMM [49,111–114]. The mitochondrial E3 ubiquitin ligase MITOL has been proposed to introduce the initial ubiquitin important for Parkin recruitment [115]. During Parkin activation, auto-inhibited Parkin binds to the phosphorylated ubiquitin, thus translocating to damaged mitochondria, where the mentioned structural rearrangements activate Parkin. PINK1 phosphorylates both monomeric ubiquitin and poly-ubiquitin chains that Parkin binds to, hence retaining Parkin on the OMM. This binding of Parkin to the OMM boosts the formation of additional mono- and poly-ubiquitinated substrate proteins, initiating a positive feedback loop in which PINK1 phosphorylates additional Parkin and ubiquitin molecules to activate and retain additional Parkin on the surface of dysfunctional mitochondria [49,116]. Furthermore, recent studies indicate Parkin activation independent of Parkin phosphorylation by PINK1, supporting the feed-forward mechanism and rapid degradation of mitochondria through the Parkin-PINK1 mitophagy pathway. In this scenario, the phosphor-binding pocket in the RING0 domain of Parkin binds to phosphorylated ubiquitin on the OMM, releasing the catalytic RING2 from the auto-inhibited position [61,62]. These findings may explain how Parkin, without its UBL domain, retains some ability to induce mitophagy [24,53,66].

**Fig. 4:**
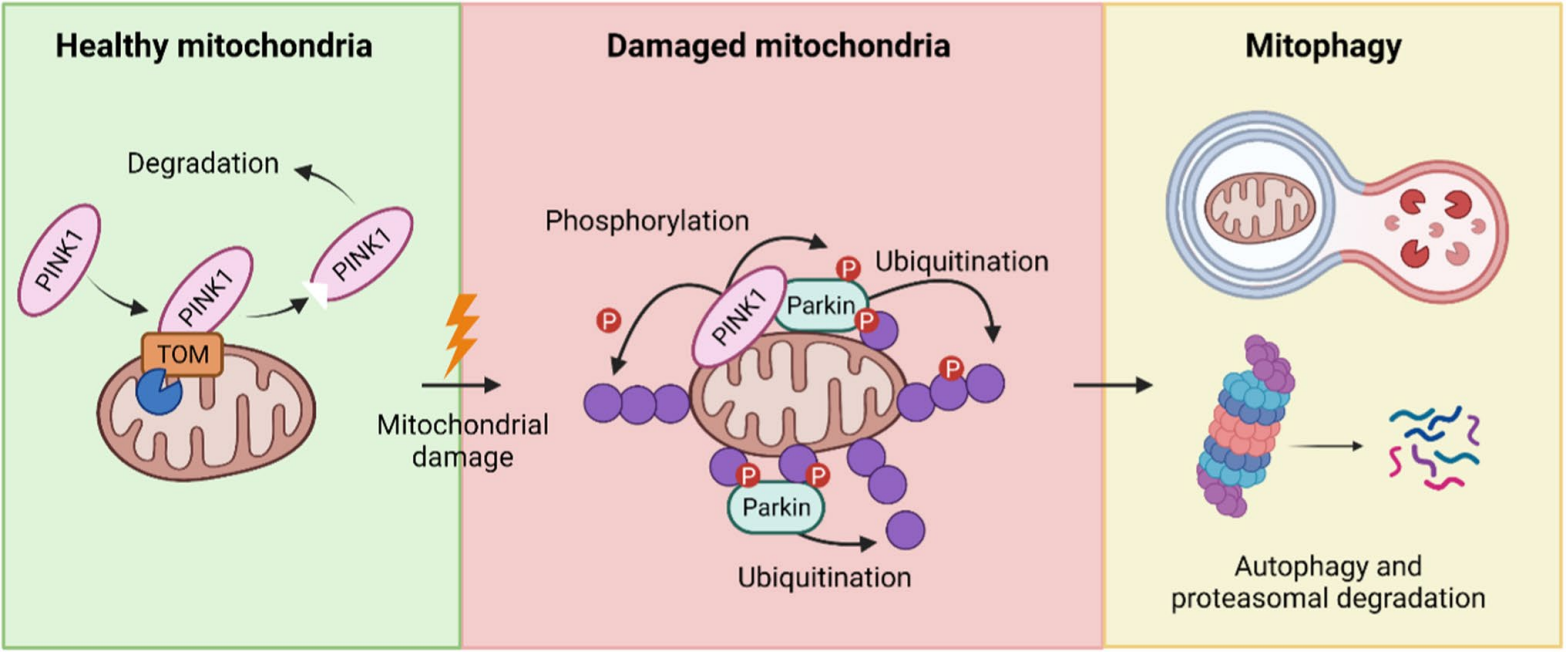
Parkin-dependent mitophagy. When mitochondria are healthy PINK1 is translocated through TOM and cleaved by the mitochondrial intramembrane protease PARL (blue pac-man) and retrotranslocated from the mitochondria to the cytosol where it is degraded. Upon mitochondrial damage, PINK1 is stabilized at the outer mitochondrial membrane (OMM), where it phosphorylates (red) OMM protein such as ubiquitin (purple), causing Parkin recruitment to damaged mitochondria. Then, Parkin activation is induced by PINK1 phosphorylation and a feedforward mechanism involving phosphorylated ubiquitin. Activated Parkin then mediates the formation of ubiquitin chains on OMM proteins that directs certain OMM proteins for proteasomal degradation and some to be recognized by autophagy receptors, which leads to the sequestering of damaged mitochondria in auto-phagosomes that fuse with lysosomes. Figure created with BioRender.com

The accumulation of ubiquitinated OMM proteins marks the mitochondria for degradation through both mitophagy and the UPS [18,51,83,100,117,118]. The initiation of mitophagy likely involves the prior degradation of some ubiquitinated OMM proteins through the UPS [51,83]. Subsequently, the ubiquitin chains present on the OMM recruit autophagic cargo receptors such as sequestosome 1 (SQSTM1/p62) and optineurin (OPTN). The dysfunctional mitochondria are then encapsulated by a double-membrane vesicle known as the autophagosome and delivered to lysosomes, where the lysosomal hydrolases finally break down the mitochondria [18] (Fig. 4).

## Negative regulation of Parkin-mediated mitophagy

Considering the tight regulation of Parkin activation, it is not surprising that Parkin-mediated mitophagy is also subject to negative regulation at several stages along the PINK1-Parkin signaling axis. Accordingly, the PTEN-L isoform of the PTEN phosphatase was shown to dephosphorylate ubiquitin and Parkin [119,120], thus forcing Parkin towards the auto-inhibited conformation, blocking its mitochondrial translocation, and suppressing mitophagy.

Similar to phosphorylation, ubiquitination is a reversible post-translation modification. Deubiquitinating enzymes (DUBs), also known as ubiquitin-specific proteases (USPs), mediate this reversal by cleaving isopeptide bonds between individual ubiquitin moieties or between ubiquitin and the protein target [121,122]. Accordingly, DUBs capable of trimming the ubiquitin or phospho-ubiquitin chains on OMM proteins antagonize mitophagy, and are thus obvious drug targets for PD [123–126]. Intriguingly, USP30, a K6-ubiquitin-chain-specific DUB localized on the OMM, has been shown to antagonize Parkin/PINK1-dependent mitophagy by deubiquitinating OMM proteins, including TOM20 [127–129]. Moreover, several other DUBs [130], including ataxin-3 [131,132], USP8 [133], and USP15 [134,135], have been reported to deubiquitinate mitochondrial proteins, and some redundancy between these enzymes is expected.

## Other functions of Parkin

In addition to its role in mitochondrial quality control and PD, Parkin has been implicated in various other functions, too extensive to be discussed exhaustively here. However, notably a correlation between impaired Parkin activity and increased risk of cancer has been observed. In turn, this indicates that Parkin activity and efficient mitochondrial quality control provide a mechanism for tumor suppression [136,137]. As with this observation, most of the additional functions attributed to Parkin are still, albeit more indirectly, connected with mitochondrial homeostasis and dependent on PINK1. For instance, PINK1 and Parkin regulate PGC1α activation through degradation of the transcriptional repressor ZNF746 (also known as PARIS). PINK1 directly phosphorylates PARIS, priming it for ubiquitination by Parkin, which interacts with the C-terminal zinc finger domain of PARIS and tags it for degradation [138]. Since PGC1α stimulates the synthesis of mitochondrial DNA, protein, and membrane [139], Parkin may also in this way affect mitochondrial homeostasis. Accordingly, overexpression of PARIS leads to the loss of dopaminergic neurons, an effect that can be reversed by co-expression of either Parkin or PGC1α [138]. Studies have also shown that Parkin plays a role in ubiquitin-mediated autophagy of intracellular *Mycobacterium tuberculosis* [140], which may explain why certain non-coding *PRKN* polymorphisms have been linked to increased susceptibility to intracellular pathogens [141,142].

Finally, a link between immune signaling by the kinase TBK1 and Parkin-dependent mitophagy was recently shown to occur via OPTN [143]. Thus, after Parkin-dependent mitochondrial ubiquitination, OPTN assembly stimulates TBK1 which then further stimulates OPTN assembly [143]. This positive feedback loop eventually results in the elimination of damaged mitochondria and also connects Parkin and neurodegeneration with inflammatory processes.

## The fragility of the *PRKN* gene

The *PRKN* gene is located in the so-called FRA6E region on chromosome 6, one of the most fragile areas of the human genome [144]. Typically, chromosomal fragile sites are hotspots for deletions and amplifications, and fragility of the FRA6E region is likely caused by replication problems linked to transcription of the extremely large *PRKN* gene [144–146]. Thus, even though the mature *PRKN* mRNA is only about 4 kb, the *PRKN* gene and primary transcript is a whopping 1.4 Mb which mainly consists of introns. Since, the size of the region is conserved in vertebrates, the non-coding DNA may possess some unknown regulatory function [144,147]. A recent study has shown that the large introns do not seem to be important for *PRKN* expression ^146^, but may through the fragility of the region contribute to an increased risk of de novo germline variants in *PRKN*. Moreover, the fragility of the *PRKN* gene likely increases the risk of somatic *PRKN* mutations, which based on Parkin’s possible role as a tumor suppressor [136,137], could have implications for cancer susceptibility.

## PD-associated *PRKN* variants

To aid research and diagnosis of genetic disorders, information on gene variants and their significance to disease have been collected in databases such as ClinVar [23,148]. For certain diseases, specialized databases have been developed that focus on a single disease or a subset of diseases. In case of *PRKN* gene variants, these are recorded in both ClinVar and the Movement Disorder Society Genetic mutation database (MDSGene) [149]. Currently, ClinVar comprises information on more than 650 different *PRKN* variants reported and interpreted by clinical and research laboratories as either “benign”, “likely benign”, “likely pathogenic”, “pathogenic”, “conflicting interpretations” or “variant of uncertain significance (VUS)”. In addition to the clinical status of the reported *PRKN* variants, the databases also include information on the type of variant. Synonymous variants were once perceived as “silent” with no impact on protein abundance, structure and activity, but some synonymous variants have later been demonstrated to partake in various human diseases by the deletion or creation of splice sites, or by affecting RNA stability, miRNA targeting or the rate of translation, thus altering co-translational protein folding [150]. However, in general synonymous variants only rarely affect function, and in the case of *PRKN* none of the reported synonymous variants are classified as disease-linked in ClinVar or MDSGene [151]. From a total of 1350 *PRKN* sequence variants reported in MDSGene, most (43.5%) of the disease-linked variants are so-called structural variants causing exon rearrangements. These are followed by missense (22.3%), frameshift (16.5%), nonsense (7.9%), and splice-site variants (7.9%) [151]. Structural and nonsense variants are typically highly detrimental and are therefore generally predicted to cause loss of function. In contrast, the effect of missense variants can range from subtle to dramatic impact on protein structure, stability and activity, dependent on the position and nature of the amino acid substitution. Accordingly, predicting the impact of missense variants on protein stability and function is not straightforward, and missense *PRKN* variants therefore represent the largest group that is clinically classified as conflicting or as variants of unknown significance (VUS).

## Inactivating mechanisms of disease-linked Parkin missense variants

**Fig. 5:**
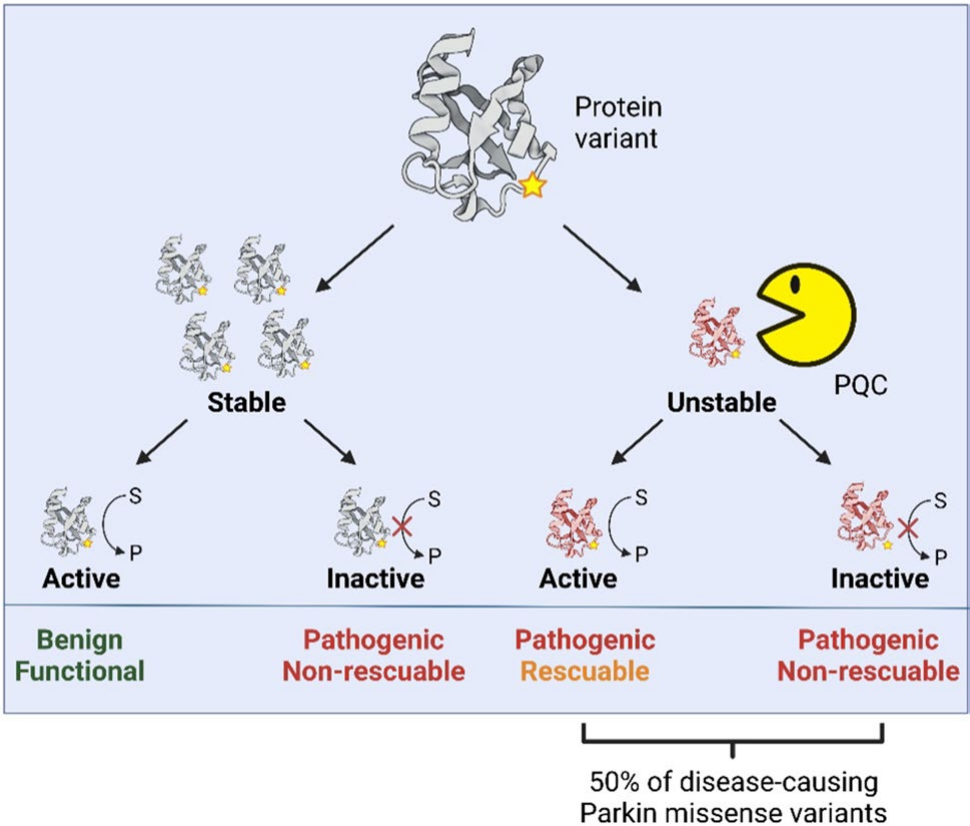
Possible mechanisms for Parkin inactivation by missense variants. Variant interpretation may elucidate the specific disease mechanism of a given variant and help identify gene variants that can potentially be rescued. Figure created with BioRender.com

Missense variants can inhibit gene function in multiple ways. However, two common mechanisms, which are not mutually exclusive, include either a direct ablation of protein activity e.g. by disruption of the active site, or indirectly by destabilizing the native fold (Fig. 5) [152]. In some cases, the position and nature of the substitution may provide insights into its mechanism of pathogenicity. Thus, Parkin missense variants that directly inhibit the catalytic activity are expected to occur at (or near) the catalytic C431 residue [54,153–155]. The pathogenic variants: T415N, G430D and P437L are all positioned close to the active site and were found to be expressed at high levels but displayed very low mitophagy upon mitochondrial damage [25], indicating that they may alter the catalytic site causing impaired functionality without strongly affecting Parkin folding and structural stability. The Parkin M1T and M1V variants, affecting the start codon, will disturb translation initiation. The consequence of using an alternative in-frame start codon found at position 80 would result in a version of Parkin lacking its UBL domain [156], which has been observed in human cell lines [155]. Despite the mentioned report that Parkin lacking its UBL domain is functional, the M1T and M1V variants are pathogenic. Another variant, K161N, found in a family with PD [157], affects a position involved in binding the phosphorylated UBL to the RING0 domain and abolishes Parkin’s ability to mediate mitophagy [25,158], emphasizing the importance of this residue and interaction for Parkin function.

Disease-linked missense variants located outside the active site or other critical functional positions are likely to affect protein folding or stability, thus more indirectly inhibiting function. Typically, structurally destabilized proteins are prone to interact with components of the cellular protein quality control (PQC) system, including molecular chaperones and components of the UPS, leading to rapid PQC-linked degradation and reduced abundance of the missense protein. For instance, *PRKN* variants affecting the zinc coordinating residues, such as the PD-associated variants, C212Y, C253Y and C441R, are of low abundance and display impaired capability to induce mitophagy in response to mitochondrial stress [25]. In addition, the well-described pathogenic variant R42P [21,159] introduces a steric clash in the UBL domain, causing global unfolding of the UBL domain and drastic loss of protein abundance due to its rapid proteasomal degradation [25,155,160,161]. However, as the active site in RING2 is intact, the R42P variant may still be enzymatically active. Indeed, Parkin R42P has been shown to retain function [25,53,158,162,163]. Accordingly, using the Muller classification of mutations [164], R42P is an example of a hypomorph variant (a variant with reduced activity), while an active site variant would display a complete loss of function and thus be categorized as amorphic. Obviously, also stable variants may retain some enzymatic function and far from all destabilized variants are expected to be hypomorphs, since some variants may affect both activity and structural stability of the protein, which in case of Parkin will likely include substitutions of residues coordinating the Zn^2+^ ions in RING2.

## Potential for restoring Parkin variant abundance and activity

The distinction between hypomorphic and amorphic missense variants is important, since hypomorphic alleles can, in principle, be rescued, whereas amorphic variants, similar to a deletion, cannot. Thus, increasing the abundance of a hypomorphic Parkin variant above some critical threshold could result in reactivation of mitophagy and thus mitigate disease. Potentially, this could be accomplished in multiple ways, such as through small molecule stabilizers, or by boosting synthesis or blocking the PQC-linked degradation. Though this has yet to be attempted for Parkin, based on other genetic disorders, these strategies hold some promise. For instance, small molecule stabilizers of the CFTR protein have proven effective for cystic fibrosis [165–167], and in the case of the hereditary cancer predisposition disease known as Lynch syndrome, blocking PQC-linked degradation restores function and drug sensitivity in cell models [168,169].

Currently, it is unknown how many of the Parkin missense variants are hypomorphic. However, recent estimates based on computational studies and high-throughput experiments have shown that as much as 60% of disease-linked missense variants results in significantly reduced structural stability of the encoded protein [152,170], which in turn is expected to lead to PQC-mediated degradation and a reduced abundance of the protein [171,172]. Based on recent deep mutational scanning of the abundance of > 99% of all possible Parkin variants, 50% of the known disease-linked Parkin missense variants were found to result in dramatically reduced steady-state levels (Fig. 5) [163]. Among all possible Parkin missense variants, 40% displayed an abundance reduced by more than half the amount of wild-type Parkin [163], and at least some of these are likely to be hypomorphic. Indeed, since Parkin is a multi-domain protein, it is likely that many low abundance missense variants positioned outside the catalytic RING2 domain are hypomorphic similar to R42P. However, the modular nature of the Parkin structure also challenges the development of stabilizing small molecules, since likely such a molecule would only correct protein folding/stability locally in the domain to which it binds. Accordingly, studies on enhancing *PRKN* expression and blocking the degradation of destabilized Parkin variants are warranted.

## Degradation of destabilized Parkin variants

Since evolution selects for function rather than stability, most proteins are not overly stable under physiological conditions [173–175], and are therefore susceptible to degradation under stress conditions. Accordingly, various stressors can cause a decrease in Parkin protein levels [176–180]. In addition, Parkin loss is also observed upon mitochondrial depolarization, through either Parkin-dependent mitophagy or auto-ubiquitination and proteasomal degradation [133,181–184]. A more recent study found that Parkin is targeted for proteasomal degradation in response to stress conditions, independently of auto-ubiquitination and mitophagy [176].

As protein folding and structural stability are determined by the amino acid sequence [185], missense variants may, similar to a stress situation, lead to a reduced thermodynamic stability of the protein structure. Accordingly, a larger fraction of such proteins will, relatively to the wild-type, be found in a partially or fully unfolded conformation, and are therefore prone to form non-specific interactions with other cell components, thus poisoning the intracellular environment. To mitigate this danger, cells are equipped with a protein quality control (PQC) system that catalyzes the refolding or degradation of these aberrant proteins [172,186]. Molecular chaperones promote protein folding [187,188], but also collaborate with ubiquitin-protein ligases and the proteasome to clear non-native proteins from the cytosol and nucleus [189–193].

It is well known, that certain pathogenic *PRKN* variants may lead to a structural destabilization of the protein, which in turn results in reduced solubility, aggregate formation, and increased degradation [155,194,195]. It is therefore not surprising that such Parkin variants are subject to regulation by the PQC system. For instance, Hsp70 overexpression has been observed to ameliorate phenotypes of *Drosophila* Parkin mutants [196]. In addition, increased expression of chaperones has been found to both prevent aggregation of wild-type Parkin and promote the folding of the W453stop nonsense variant [197], while the J-domain Hsp70 co-chaperone DNAJB2 has been found to restore function to the low-abundance C289G Parkin variant [194]. Conversely, BAG5, another Hsp70 co-chaperone, has been found to interact with Parkin, inhibit its ubiquitin-ligase activity and enhance Parkin localization in protein aggregates [198]. Accordingly, molecular chaperones have been suggested as targets for PD therapeutics [199,200].

**Fig. 6:**
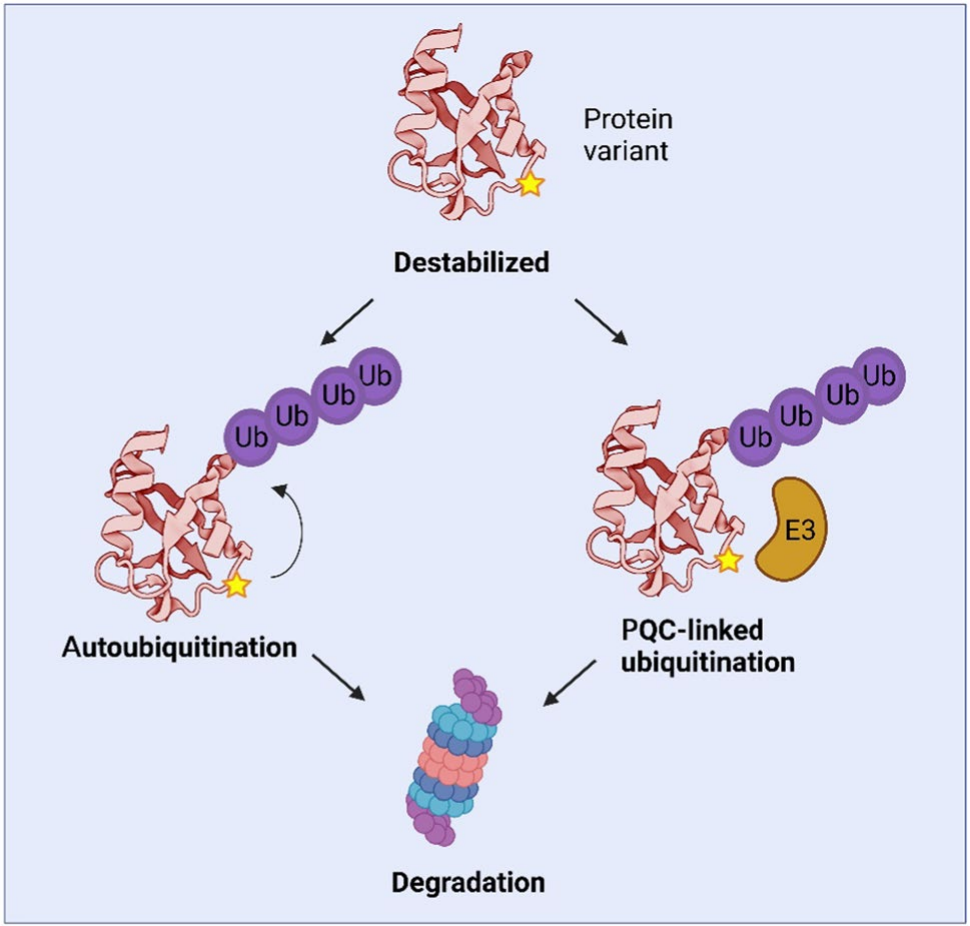
Ubiquitination and proteasomal degradation of destabilized Parkin variants. Parkin, which is structurally destabilized due to a missense variant (star), can be subject to both increased auto-ubiquitination and PQC-linked ubiquitination by other cellular E3s (orange). Figure created with BioRender.com

In addition to PQC-mediated degradation of destabilized Parkin variants, certain Parkin missense variants in the N-terminal UBL domain such as R42P have been shown to disrupt the auto-inhibited conformation, leading to Parkin activation and auto-ubiquitination, followed by degradation [155,160,161,163]. Hence, unlike most PQC targets, structural destabilization of Parkin can trigger two separate mechanisms of Parkin ubiquitination (Fig. 6). However, importantly increased auto-ubiquitination does not preclude PQC-mediated ubiquitination, and the auto-ubiquitination-dependent clearance of Parkin variants will only apply to the subset of destabilized Parkin variants that are hypomorphs.

**Fig. 7:**
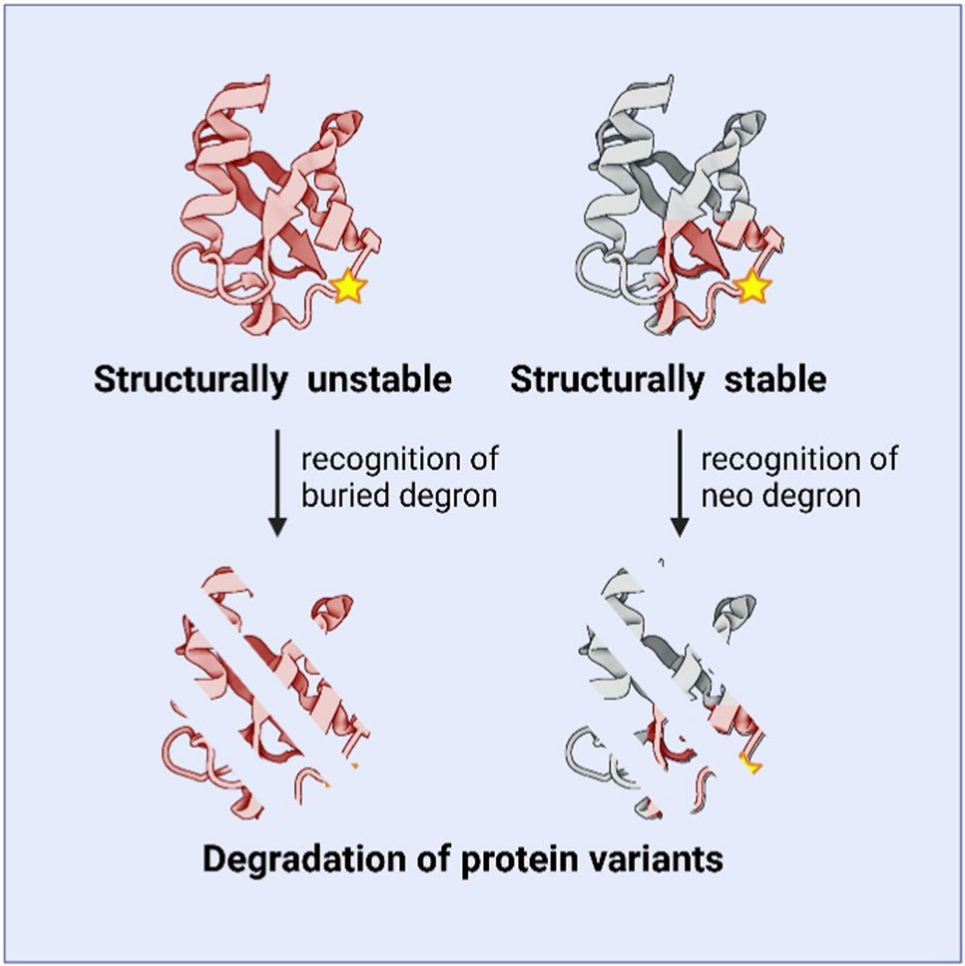
The role of degrons in the degradation of Parkin variants. Structurally destabilized Parkin variants may transiently expose buried PQC degrons leading to recognition by PQC components and degradation (left). Alternatively, surface exposed degrons may be generated as a direct result of a missense variant (yellow star). Such neo degrons also lead to degradation of the protein variant. However, in this case the protein is structurally stable and likely still functional (right). Figure created with BioRender.com

In PQC-dependent protein degradation, recent data suggest that the discriminating feature recognized by the degradation system is the exposure of degradation signals (degrons) through local or global unfolding events [171,172,201,202]. It has recently been shown that many such quality control degrons are enriched in hydrophobic residues and depleted for negatively charged residues [203–208]. Accordingly, these degrons are typically buried inside the native protein structure but become exposed upon unfolding or misfolding [201,209]. In a recent systematic study, we found several such PQC degrons are found within all of the structured domains in Parkin [163]. Presumably, upon introduction of a destabilizing amino acid substitution, local or global unfolding will result in exposure of some of these PQC degrons that in turn will recruit molecular chaperones and PQC E3s to facilitate ubiquitination and degradation (Fig. 7).

An alternative mechanism, by which amino acid substitutions may trigger degradation, is by generating degrons in exposed regions of the protein. In this situation, the amino acid substitution need not destabilize the native fold, since the degron is surface exposed and thus can be directly recognized by the E3. Although none of the known disease-linked Parkin variants have been shown to operate in this manner, specific missense variants in the disordered linker region between the UBL and RING0 domains can form such a neo-degron [163] (Fig. 7). As the region is unstructured, the prediction is that such degron forming variants will not affect the structural stability or function of the protein, and degron generating variants should therefore be hypomorphic. However, as the region is conserved through evolution and proximal to the ACT element involved in Parkin activation [40], it is possible that the degron formation via amino acid substitutions at this position is an indirect consequence of the sequence properties required for function of the ACT region. Thus, the functional requirement for an exposed, partially hydrophobic motif makes the ACT region prone to missense variants that introduce additional hydrophobic residues and the formation of a neo-degron.

Interestingly, in depth studies have shown that some Parkin missense variants, including V224A, W403A and F146A enhance Parkin-dependent mitophagy [24,25,210]. In addition, these were shown to rescue mitophagy when expressed *in cis* with several otherwise non-functional variants [25] and thus appear to function as intragenic second site suppressors of certain disease-linked Parkin variants, including variants in regions important for PINK1-mediated activation or E2 binding sites such as K211N and T240M, respectively, as well as low abundance variants such as R42P, V56E and R275W. Mechanistically, such suppressors could operate by increasing the specific enzyme activity, increasing phospho-ubiquitin binding, mitochondrial translocation, and/or by stabilizing the native conformation, thus increasing Parkin abundance. However, since several of the hyperactive variants actually appear to destabilize the closed inactive Parkin conformation [25,210], they likely operate by shifting Parkin towards the active conformation. In turn, this may lead to increased autoubiquitination and/or a general destabilization of the Parkin structure. This is supported by the observation that some of these variants display reduced steady-state levels [163], and therefore, paradoxically the hyperactive Parkin variants could also be hypomorphic. Hopefully, future studies will probe these observations further, which could potentially pave the way for developing therapeutics mimicking the second site suppressors.

## Variants of uncertain significance

**Fig. 8:**
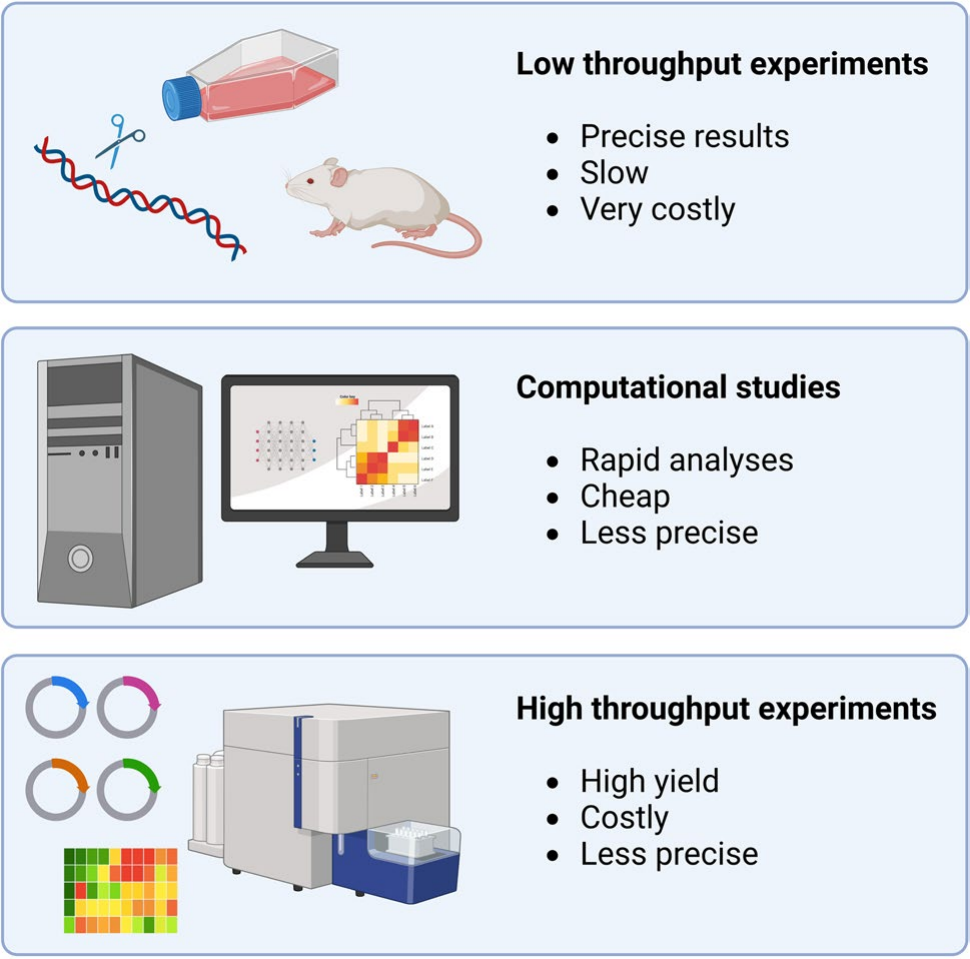
Approaches to classification of variant effects. The effects of *PRKN* gene variants can be assessed by traditional and highly detailed, but often very costly low throughput approaches (upper panel). Computational studies offer an alternative approach, which is cheap and rapid, but also sometimes less precise (middle panel). Finally, high-throughput experimental approaches allow libraries of gene variants to be assayed in large, multiplexed experiments (lower panel). Figure created with BioRender.com

Like most severe monogenic disorders, *PRKN*-linked PD is rare. However, due to the increased speed and lowered cost of DNA sequencing, a growing number of individuals are being sequenced and diagnosed with *PRKN*-linked PD and other genetic diseases. In addition, the rise in genome sequencing also results in an increased number of newly identified gene variants, whose pathogenicity must be carefully determined. For instance, whole exome sequencing analyses return ~ 50,000 autosomal variants per individual compared to a reference genome [211]. Only a small fraction will cause monogenic disease while the majority are either harmless or may contribute to multifactorial disease. However, often there is insufficient evidence to classify newly observed variants as being either benign or pathogenic and instead these are designated as VUS [212]. In terms of diagnosis and genetic counseling of affected individuals or families, clinical sequencing is only relevant if variants are classified, which renders the large number of VUS highly problematic. To improve clinical diagnostic yield from genome sequencing there is a need to increase the speed of variant classification. As evident from the above, variant classification, in particular of missense variants, is not straightforward. While population-based studies are doing well at linking common variants with phenotypes, they require many individuals and are often inadequate in the case of rare variants [212]. Traditionally, the effect of a VUS is assessed in a low-throughput manner (Fig. 8), efforts that can take several months or years. Though such assays typically yield detailed and highly accurate results, this approach is not feasible considering the number of observed gene variants. Indeed, given the size of the human genome, the mutation rate, and the current global population, all single nucleotide variants compatible with life are likely to exist in the current human population [213]. For this reason, there is a need for assessing not only already observed variants, but essentially all variants [214], including the rare variants that have not yet been observed in the population.

To approach this issue, both computational techniques and laboratory-based high-throughput assays have been developed to rapidly generate variant effect data. Computational models typically assess the impact of gene variants based on sequence conservation and/or on structural data, and models are trained to predict the variant effects on protein function. These in silico predictors provide a non-laborious and fast method to predict the impact of variants making them attractive (Fig. 8). With recent developments, precise variant effect predictions are possible [215,216], at least for the kinds of variants, genes and diseases for which we can accurately assess such pathogenicity predictions. However, in general computational tools do not inform on the mechanistic basis of the variant effects. The laboratory-based high-throughput assays, though demanding, offer an approach to variant classification [214] (Fig. 8), and generate data, which may also be useful for training future computer-based predictors. Our recent deep mutational scanning of Parkin variant abundance revealed that structural destabilization and degradation explain about half of the pathogenic Parkin variants [163], and is thus on its own, not highly useful for variant classification. Accordingly, additional comprehensive assessments of variant effects are required, such as high-content mapping of Parkin function e.g. by monitoring Parkin’s ability to initiate mitophagy.

## Concluding remarks

Mitochondrial biogenesis, mitochondrial dynamics, and mitophagy form a continuous process to preserve optimal mitochondrial quality. Parkin, as a key protein, plays a pivotal role in the entire spectrum of mitochondrial quality control.

As evident from the above, studying the cellular and molecular functions of Parkin provides important insights into not only the monogenic forms of PD, but also indirectly increases our understanding of idiopathic PD. More specifically, studies on Parkin variant effects are useful for clinical genetics and variant classification, but also benefit our understanding of the structural, biophysical and cellular impact of gene variants, regardless of their frequency in the population [217].

Although variant classification based on high-throughput experimental or computational data is currently showing promising progression, these methods will still wrongly classify some variants and also do not provide highly detailed information on the molecular and cellular mechanisms involved. Meticulous low-throughput structural, biophysical, and animal studies are therefore still exceedingly important. This is exemplified by the studies showing second site suppressors of certain disease-linked Parkin variants [25,210], which may guide development of small molecules with similar effects. Recently, a positive allosteric modulator of Parkin activity, BIO-2007817, was described [218], but the compound did not increase mitophagy [125,218] and did not broadly stabilize low abundant Parkin variants in cell-based studies [163]. However, other small molecules that interact with Parkin could potentially activate Parkin or at least stabilize the protein and thus increase the intracellular amount of low abundance pathogenic variants [171,219]. Alternatively, increased cellular Parkin levels could also be achieved by boosting Parkin synthesis or blocking Parkin degradation, and for this reason further studies on Parkin proteostasis are required. For instance, although some chaperones and co-chaperones have been shown to triage Parkin variants [194,196–198], it is unknown if these engage broadly with structurally destabilized disease-linked variants. In addition, the identity of the E2 and E3 enzymes involved in the PQC-linked proteasomal degradation of structurally unstable Parkin variants are still unknown, despite their potential importance as drug targets for low-abundance variants. Other approaches to activate Parkin-mediated mitophagy are currently ongoing and include USP30 inhibitors [220] or activators of PINK1 or ULK1 (recently reviewed [125]). Importantly, such therapeutics may be relevant not only for *PRKN*-linked PD, but likely also for idiopathic PD.

## Data Availability

Not applicable.

## References

[1] Poewe W (2017). Parkinson disease. Nat Rev Dis Primers.

[2] Balestrino R, Schapira AHV (2020). Parkinson disease. Eur J Neurol.

[3] Vázquez-Vélez GE, Zoghbi HY (2021). Parkinson’s disease genetics and pathophysiology. Annu Rev Neurosci.

[4] Cheng HC, Ulane CM, Burke RE (2010). Clinical progression in Parkinson disease and the neurobiology of axons. Ann Neurol.

[5] Armstrong MJ, Okun MS (2020). Diagnosis and treatment of Parkinson disease: a review. JAMA.

[6] Goedert M (2001). Alpha-synuclein and neurodegenerative diseases. Nat Rev Neurosci.

[7] Savitt JM, Dawson VL, Dawson TM (2006). Diagnosis and treatment of Parkinson disease: molecules to medicine. J Clin Invest.

[8] East DA, Campanella M (2016). Mitophagy and the therapeutic clearance of damaged mitochondria for neuroprotection. Int J Biochem Cell Biol.

[9] Chan DC (2020). Mitochondrial dynamics and its involvement in disease. Annu Rev Pathol.

[10] William Langston J, Ballard P, Tetrud JW, Irwin I (1983). Chronic Parkinsonism in humans due to a product of meperidine-analog synthesis. Science.

[11] Park JS, Davis RL, Sue CM (2018). Mitochondrial Dysfunction in Parkinson’s Disease: New Mechanistic Insights and Therapeutic Perspectives. Curr Neurol Neurosci Rep.

[12] Pickrell AM, Youle RJ (2015). The roles of PINK1, parkin, and mitochondrial fidelity in Parkinson’s disease. Neuron.

[13] Bandres-Ciga S, Diez-Fairen M, Kim JJ, Singleton AB (2020). Genetics of Parkinson’s disease: an introspection of its journey towards precision medicine. Neurobiol Dis.

[14] Nalls MA (2019). Identification of novel risk loci, causal insights, and heritable risk for Parkinson’s disease: a meta-analysis of genome-wide association studies. Lancet Neurol.

[15] Truban D, Hou X, Caulfield TR, Fiesel FC, Springer W (2017). PINK1, Parkin, and mitochondrial quality control: what can we learn about Parkinson’s disease pathobiology?. J Parkinsons Dis.

[16] Bayne AN, Trempe JF (2019). Mechanisms of PINK1, ubiquitin and Parkin interactions in mitochondrial quality control and beyond. Cell Mol Life Sci.

[17] Trempe JF, Gehring K (2023). Structural mechanisms of mitochondrial quality control mediated by PINK1 and parkin. J Mol Biol.

[18] Wade Harper J, Ordureau A, Heo JM (2018). Building and decoding ubiquitin chains for mitophagy. Nat Rev Mol Cell Biol.

[19] Matsuda N, Yamano K (2020). Two sides of a coin: Physiological significance and molecular mechanisms for damage-induced mitochondrial localization of PINK1 and Parkin. Neurosci Res.

[20] Tanaka K (2020). The PINK1-Parkin axis: an overview. Neurosci Res.

[21] Shimura H (2000). Familial Parkinson disease gene product, parkin, is a ubiquitin-protein ligase. Nat Genet.

[22] Kitada T (1998). Mutations in the parkin gene cause autosomal recessive juvenile Parkinsonism. Nature.

[23] Landrum MJ (2020). ClinVar: improvements to accessing data. Nucleic Acids Res.

[24] Tang MY (2017). Structure-guided mutagenesis reveals a hierarchical mechanism of Parkin activation. Nat Commun.

[25] Yi W (2019). The landscape of Parkin variants reveals pathogenic mechanisms and therapeutic targets in Parkinson’s disease. Hum Mol Genet.

[26] Brüggemann N Klein, C. Parkin Type of Early-Onset Parkinson Disease. GeneReviews® (2020).

[27] Lesage S (2020). Characterization of recessive Parkinson disease in a large multicenter study. Ann Neurol.

[28] Lücking CB (2000). Association between early-onset Parkinson’s disease and mutations in the parkin gene. N Engl J Med.

[29] Corti O, Lesage S, Brice A (2011). What genetics tells us about the causes and mechanisms of Parkinson’s disease. Physiol Rev.

[30] Wasner K, Grünewald A, Klein C (2020). Parkin-linked Parkinson’s disease: From clinical insights to pathogenic mechanisms and novel therapeutic approaches. Neurosci Res.

[31] Mori H (1998). Pathologic and biochemical studies of juvenile parkinsonism linked to chromosome 6q. Neurology.

[32] Bogetofte H (2019). PARK2 mutation causes metabolic disturbances and impaired survival of human iPSC-derived neurons. Front Cell Neurosci.

[33] Bogetofte H (2019). Perturbations in RhoA signalling cause altered migration and impaired neuritogenesis in human iPSC-derived neural cells with PARK2 mutation. Neurobiol Dis.

[34] Okarmus J (2020). Lysosomal perturbations in human dopaminergic neurons derived from induced pluripotent stem cells with PARK2 mutation. Sci Rep.

[35] Okarmus J (2021). Identification of bioactive metabolites in human iPSC-derived dopaminergic neurons with PARK2 mutation: altered mitochondrial and energy metabolism. Stem Cell Rep.

[36] Wei PC (2019). Neuroprotection of indole-derivative compound NC001–8 by the regulation of the NRF2 pathway in Parkinson’s disease cell models. Oxid Med Cell Longev.

[37] Shaltouki A (2015). Mitochondrial alterations by PARKIN in dopaminergic neurons using PARK2 patient-specific and PARK2 knockout isogenic iPSC lines. Stem Cell Reports.

[38] Imaizumi Y (2012). Mitochondrial dysfunction associated with increased oxidative stress and α-synuclein accumulation in PARK2 iPSC-derived neurons and postmortem brain tissue. Mol Brain.

[39] Seirafi M, Kozlov G, Gehring K (2015). Parkin structure and function. FEBS J.

[40] Gladkova C, Maslen SL, Skehel JM, Komander D (2018). Mechanism of parkin activation by PINK1. Nature.

[41] Trempe JF (2013). Structure of parkin reveals mechanisms for ubiquitin ligase activation. Science.

[42] Hershko A, Ciechanover A (1998). The ubiquitin system. Annu Rev Biochem.

[43] Zheng N, Shabek N (2017). Ubiquitin ligases: structure, function, and regulation. Annu Rev Biochem.

[44] Varshavsky A (2017). The ubiquitin system, autophagy, and regulated protein degradation. Annu Rev Biochem.

[45] Kwon YT, Ciechanover A (2017). The ubiquitin code in the ubiquitin-proteasome system and autophagy. Trends Biochem Sci.

[46] Wenzel DM, Lissounov A, Brzovic PS, Klevit RE (2011). UBCH7 reactivity profile reveals parkin and HHARI to be RING/HECT hybrids. Nature.

[47] Dye BT, Schulman BA (2007). Structural mechanisms underlying posttranslational modification by ubiquitin-like proteins. Annu Rev Biophys Biomol Struct.

[48] Wenzel DM, Klevit RE (2012). Following Ariadne’s thread: a new perspective on RBR ubiquitin ligases. BMC Biol.

[49] Ordureau A (2014). Quantitative proteomics reveal a feedforward mechanism for mitochondrial pARKIN translocation and ubiquitin chain synthesis. Mol Cell.

[50] Ordureau A (2020). Global landscape and dynamics of Parkin and USP30-dependent ubiquitylomes in ineurons during mitophagic signaling. Mol Cell.

[51] Chan NC (2011). Broad activation of the ubiquitin-proteasome system by Parkin is critical for mitophagy. Hum Mol Genet.

[52] Walden H, Martinez-Torres RJ (2012). Regulation of Parkin E3 ubiquitin ligase activity. Cell Mol Life Sci.

[53] Chaugule VK (2011). Autoregulation of Parkin activity through its ubiquitin-like domain. EMBO J.

[54] Wauer T, Komander D (2013). Structure of the human Parkin ligase domain in an autoinhibited state. EMBO J.

[55] Kumar A (2017). Parkin-phosphoubiquitin complex reveals cryptic ubiquitin-binding site required for RBR ligase activity. Nat Struct Mol Biol.

[56] McWilliams TG (2018). Phosphorylation of Parkin at serine 65 is essential for its activation in vivo. Open Biol.

[57] Kumar A (2015). Disruption of the autoinhibited state primes the E3 ligase parkin for activation and catalysis. EMBO J.

[58] Wauer T, Simicek M, Schubert A, Komander D (2015). Mechanism of phospho-ubiquitin-induced PARKIN activation. Nature.

[59] Sauvé V (2015). A Ubl/ubiquitin switch in the activation of Parkin. EMBO J.

[60] Sauvé V (2018). Mechanism of parkin activation by phosphorylation. Nat Struct Mol Biol.

[61] Sauvé V (2022). Structural basis for feedforward control in the PINK1/Parkin pathway. EMBO J.

[62] Fakih R, Sauvé V, Gehring K (2022). Structure of the second phosphoubiquitin-binding site in parkin. J Biol Chem.

[63] Kondapalli C (2012). PINK1 is activated by mitochondrial membrane potential depolarization and stimulates Parkin E3 ligase activity by phosphorylating Serine 65. Open Biol.

[64] Iguchi M (2013). Parkin-catalyzed ubiquitin-ester transfer is triggered by PINK1-dependent phosphorylation. J Biol Chem.

[65] Schubert AF (2017). Structure of PINK1 in complex with its substrate ubiquitin. Nature.

[66] Shiba-Fukushima K (2012). PINK1-mediated phosphorylation of the Parkin ubiquitin-like domain primes mitochondrial translocation of Parkin and regulates mitophagy. Sci Rep.

[67] Herst PM, Rowe MR, Carson GM, Berridge MV (2017). Functional mitochondria in health and disease. Front Endocrinol (Lausanne).

[68] Friedman JR, Nunnari J (2014). Mitochondrial form and function. Nature.

[69] Scheibye-Knudsen M, Fang EF, Croteau DL, Wilson DM, Bohr VA (2015). Protecting the mitochondrial powerhouse. Trends Cell Biol.

[70] Cheng YW, Liu J, Finkel T (2023). Mitohormesis. Cell Metab.

[71] Karbowski M, Oshima Y, Verhoeven N (2022). Mitochondrial proteotoxicity: implications and ubiquitin-dependent quality control mechanisms. Cell Mol Life Sci.

[72] Liu S, Liu S, Jiang H (2022). Multifaceted roles of mitochondrial stress responses under ETC dysfunction—repair, destruction and pathogenesis. FEBS J.

[73] Ploumi C, Daskalaki I, Tavernarakis N (2017). Mitochondrial biogenesis and clearance: a balancing act. FEBS J.

[74] Gottlieb RA (2021). At the heart of mitochondrial quality control: many roads to the top. Cell Mol Life Sci.

[75] Shen Y (2023). Mitochondrial dynamics in neurological diseases: a narrative review. Ann Transl Med.

[76] Liu X, Weaver D, Shirihai O, Hajnóczky G (2009). Mitochondrial ‘kiss-and-run’’: interplay between mitochondrial motility and fusion–fission dynamics’. EMBO J.

[77] Wolf DM et al (2019) Individual cristae within the same mitochondrion display different membrane potentials and are functionally independent. EMBO J 38:e101056 10.15252/embj.2018101056 PMC6856616 31609012

[78] Loriette V (2023). Dynamics of mitochondrial membranes under photo-oxidative stress with high spatiotemporal resolution. Front Cell Dev Biol.

[79] Twig G (2008). Fission and selective fusion govern mitochondrial segregation and elimination by autophagy. EMBO J.

[80] Wang X (2011). PINK1 and Parkin target miro for phosphorylation and degradation to arrest mitochondrial motility. Cell.

[81] Shlevkov E, Kramer T, Schapansky J, Lavoie MJ, Schwarz TL (2016). Miro phosphorylation sites regulate Parkin recruitment and mitochondrial motility. Proc Natl Acad Sci U S A.

[82] Gegg ME (2010). Mitofusin 1 and mitofusin 2 are ubiquitinated in a PINK1/parkin-dependent manner upon induction of mitophagy. Hum Mol Genet.

[83] Tanaka A (2010). Proteasome and p97 mediate mitophagy and degradation of mitofusins induced by Parkin. J Cell Biol.

[84] Buhlman L (2014). Functional interplay between Parkin and Drp1 in mitochondrial fission and clearance. Biochim Biophys Acta.

[85] Pryde KR, Smith HL, Chau KY, Schapira AHV (2016). PINK1 disables the anti-fission machinery to segregate damaged mitochondria for mitophagy. J Cell Biol.

[86] Xu J, Deng Z, Shang S, Wang C, Han H (2023). FUNDC1 collaborates with PINK1 in regulating mitochondrial Fission and compensating for PINK1 deficiency. Biochem Biophys Res Commun.

[87] Chen X, Wang Q, Li S, Li XJ, Yang W (2022). Mitochondrial-Dependent and Independent Functions of PINK1. Front Cell Dev Biol.

[88] Burman JL (2017). Mitochondrial fission facilitates the selective mitophagy of protein aggregates. J Cell Biol.

[89] Greene JC (2003). Mitochondrial pathology and apoptotic muscle degeneration in Drosophila parkin mutants. Proc Natl Acad Sci U S A.

[90] Clark IE (2006). Drosophila pink1 is required for mitochondrial function and interacts genetically with parkin. Nature.

[91] Vincow ES (2013). The PINK1-Parkin pathway promotes both mitophagy and selective respiratory chain turnover in vivo. Proc Natl Acad Sci U S A.

[92] Valente EM (2004). Hereditary early-onset Parkinson’s disease caused by mutations in PINK1. Science.

[93] Kilarski LL (2012). Systematic review and UK-based study of PARK2 (parkin), PINK1, PARK7 (DJ-1) and LRRK2 in early-onset Parkinson’s disease. Mov Disord.

[94] Neupert W, Herrmann JM (2007). Translocation of proteins into mitochondria. Annu Rev Biochem.

[95] Greene AW (2012). Mitochondrial processing peptidase regulates PINK1 processing, import and Parkin recruitment. EMBO Rep.

[96] Kunová N (2022). Mitochondrial Processing Peptidases-Structure, Function and the Role in Human Diseases. Int J Mol Sci.

[97] Jin SM (2010). Mitochondrial membrane potential regulates PINK1 import and proteolytic destabilization by PARL. J Cell Biol.

[98] Meissner C, Lorenz H, Weihofen A, Selkoe DJ, Lemberg MK (2011). The mitochondrial intramembrane protease PARL cleaves human Pink1 to regulate Pink1 trafficking. J Neurochem.

[99] Yamano K, Youle RJ (2013). PINK1 is degraded through the N-end rule pathway. Autophagy.

[100] Narendra D, Tanaka A, Suen DF, Youle RJ (2008). Parkin is recruited selectively to impaired mitochondria and promotes their autophagy. J Cell Biol.

[101] Michaelis JB (2022). Protein import motor complex reacts to mitochondrial misfolding by reducing protein import and activating mitophagy. Nat Commun.

[102] Jin SM, Youle RJ (2013). The accumulation of misfolded proteins in the mitochondrial matrix is sensed by PINK1 to induce PARK2/Parkin-mediated mitophagy of polarized mitochondria. Autophagy.

[103] Rubio-Peña K (2020). Mitophagy of polarized sperm-derived mitochondria after fertilization. iScience.

[104] Lazarou M, Jin SM, Kane LA, Youle RJ (2012). Role of PINK1 binding to the TOM complex and alternate intracellular membranes in recruitment and activation of the E3 ligase Parkin. Dev Cell.

[105] Okatsu K (2012). PINK1 autophosphorylation upon membrane potential dissipation is essential for Parkin recruitment to damaged mitochondria. Nat Commun.

[106] Aerts L, Craessaerts K, De Strooper B, Morais VA (2015). PINK1 kinase catalytic activity is regulated by phosphorylation on serines 228 and 402. J Biol Chem.

[107] Gan ZY (2021). Activation mechanism of PINK1. Nature.

[108] Rasool S (2022). Mechanism of PINK1 activation by autophosphorylation and insights into assembly on the TOM complex. Mol Cell.

[109] Herzig S, Shaw RJ (2017). (2017) AMPK: guardian of metabolism and mitochondrial homeostasis. Nat Rev Mol Cell Biol.

[110] Hung CM et al (2021) AMPK/ULK1-mediated phosphorylation of Parkin ACT domain mediates an early step in mitophagy. Sci Adv 7:eabg4544 10.1126/sciadv.abg4544 PMC8026119 33827825

[111] Kazlauskaite A (2014). Accelerated publication: Parkin is activated by PINK1-dependent phosphorylation of ubiquitin at Ser65. Biochem J.

[112] Koyano F (2014). (2014) Ubiquitin is phosphorylated by PINK1 to activate Parkin. Nature.

[113] Kane LA (2014). PINK1 phosphorylates ubiquitin to activate Parkin E3 ubiquitin ligase activity. J Cell Biol.

[114] Wauer T (2015). Ubiquitin Ser65 phosphorylation affects ubiquitin structure, chain assembly and hydrolysis. EMBO J.

[115] Koyano F, Yamano K, Kosako H, Tanaka K, Matsuda N (2019). Parkin recruitment to impaired mitochondria for nonselective ubiquitylation is facilitated by MITOL. J Biol Chem.

[116] Ordureau A (2015). Defining roles of PARKIN and ubiquitin phosphorylation by PINK1 in mitochondrial quality control using a ubiquitin replacement strategy. Proc Natl Acad Sci U S A.

[117] Rose CM (2016). Highly multiplexed quantitative mass spectrometry analysis of ubiquitylomes. Cell Syst.

[118] Sarraf SA (2013). Landscape of the PARKIN-dependent ubiquitylome in response to mitochondrial depolarization. Nature.

[119] Wang L, Wang J, Tang Y, Shen HM (2018). PTEN-L puts a brake on mitophagy. Autophagy.

[120] Wang L (2018). PTEN-L is a novel protein phosphatase for ubiquitin dephosphorylation to inhibit PINK1-Parkin-mediated mitophagy. Cell Res.

[121] Suresh HG, Pascoe N, Andrews B (2020). The structure and function of deubiquitinases: lessons from budding yeast. Open Biol.

[122] Clague MJ, Urbé S, Komander D (2019). Breaking the chains: deubiquitylating enzyme specificity begets function. Nat Rev Mol Cell Biol.

[123] Okarmus J (2024). (2014) USP30 inhibition induces mitophagy and reduces oxidative stress in parkin-deficient human neurons. Cell Death Dis.

[124] Lange SM, Armstrong LA, Kulathu Y (2022). Deubiquitinases: From mechanisms to their inhibition by small molecules. Mol Cell.

[125] Silvian LF (2022). PINK1/Parkin Pathway activation for mitochondrial quality control—which is the best molecular target for therapy?. Front Aging Neurosci.

[126] Tsefou E, Ketteler R (2022). Targeting deubiquitinating enzymes (DUBs) that regulate mitophagy via direct or indirect interaction with Parkin. Int J Mol Sci.

[127] Sato Y (2017). Structural basis for specific cleavage of Lys6-linked polyubiquitin chains by USP30. Nat Struct Mol Biol.

[128] Gersch M (2017). Mechanism and regulation of the Lys6-selective deubiquitinase USP30. Nat Struct Mol Biol.

[129] Bingol B (2014). (2014) The mitochondrial deubiquitinase USP30 opposes parkin-mediated mitophagy. Nature.

[130] Chakraborty J, Ziviani E (2020). Deubiquitinating enzymes in Parkinson’s disease. Front Physiol.

[131] Durcan TM, Kontogiannea M, Bedard N, Wing SS, Fon EA (2012). Ataxin-3 deubiquitination is coupled to Parkin ubiquitination via E2 ubiquitin-conjugating enzyme. J Biol Chem.

[132] Durcan TM, Fon EA (2011). Mutant ataxin-3 promotes the autophagic degradation of parkin. Autophagy.

[133] Durcan TM (2014). USP8 regulates mitophagy by removing K6-linked ubiquitin conjugates from parkin. EMBO J.

[134] Cornelissen T (2018). Deficiency of parkin and PINK1 impairs age-dependent mitophagy in Drosophila. Elife.

[135] Cornelissen T (2014). The deubiquitinase USP15 antagonizes Parkin-mediated mitochondrial ubiquitination and mitophagy. Hum Mol Genet.

[136] Wang F (2004). Parkin gene alterations in hepatocellular carcinoma. Genes Chromosomes Cancer.

[137] Bernardini JP, Lazarou M, Dewson G (2017). Parkin and mitophagy in cancer. Oncogene.

[138] Shin JH (2011). PARIS (ZNF746) repression of PGC-1α contributes to neurodegeneration in Parkinson’s disease. Cell.

[139] Finck BN, Kelly DP (2006). PGC-1 coactivators: inducible regulators of energy metabolism in health and disease. J Clin Invest.

[140] Manzanillo PS (2013). The ubiquitin ligase parkin mediates resistance to intracellular pathogens. Nature.

[141] Ali S (2006). PARK2/PACRG polymorphisms and susceptibility to typhoid and paratyphoid fever. Clin Exp Immunol.

[142] Mira MT (2004). Susceptibility to leprosy is associated with PARK2 and PACRG. Nature.

[143] Yamano K (2024). Optineurin provides a mitophagy contact site for TBK1 activation. EMBO J.

[144] Pentzold C (2018). FANCD2 binding identifies conserved fragile sites at large transcribed genes in avian cells. Nucleic Acids Res.

[145] Voutsinos V, Munk SHN, Oestergaard VH (2018). Common chromosomal fragile sites-conserved failure stories. Genes (Basel).

[146] Munk SHN, Voutsinos V, Oestergaard VH (2021). Large intronic deletion of the fragile site gene PRKN dramatically lowers its fragility without impacting gene expression. Front Genet.

[147] Hattori N, Mizuno Y (2017). Twenty years since the discovery of the parkin gene. J Neural Transm (Vienna).

[148] Landrum MJ (2018). ClinVar: improving access to variant interpretations and supporting evidence. Nucleic Acids Res.

[149] Lill CM (2016). Launching the movement disorders society genetic mutation database (MDSGene). Mov Disord.

[150] Sauna ZE, Kimchi-Sarfaty C (2011). Understanding the contribution of synonymous mutations to human disease. Nat Rev Genet.

[151] Kasten M (2018). Genotype-phenotype relations for the Parkinson’s disease genes parkin, PINK1, DJ1: MDSGene systematic review. Mov Disord.

[152] Cagiada M (2021). Understanding the origins of loss of protein function by analyzing the effects of thousands of variants on activity and abundance. Mol Biol Evol.

[153] Wang C (2005). Alterations in the solubility and intracellular localization of parkin by several familial Parkinson’s disease-linked point mutations. J Neurochem.

[154] Fiesel FC (2015). Structural and functional impact of Parkinson disease-associated mutations in the E3 ubiquitin ligase parkin. Hum Mutat.

[155] Henn IH, Gostner JM, Lackner P, Tatzelt J, Winklhofer KF (2005). Pathogenic mutations inactivate parkin by distinct mechanisms. J Neurochem.

[156] Zhang BR (2010). Mutation analysis of parkin and PINK1 genes in early-onset Parkinson’s disease in China. Neurosci Lett.

[157] Abbas N (1999). A wide variety of mutations in the parkin gene are responsible for autosomal recessive parkinsonism in Europe. French Parkinson’s Disease Genetics Study Group and the European Consortium on Genetic Susceptibility in Parkinson’s Disease. Hum Mol Genet.

[158] Sriram SR (2005). Familial-associated mutations differentially disrupt the solubility, localization, binding and ubiquitination properties of parkin. Hum Mol Genet.

[159] Terreni L, Calabrese E, Calella AM, Forloni G, Mariani C (2001). New mutation (R42P) of the parkin gene in the ubiquitinlike domain associated with Parkinsonism. Neurology.

[160] Safadi SS, Shaw GS (2007). A disease state mutation unfolds the parkin ubiquitin-like domain. Biochemistry.

[161] Hampe C, Ardila-Osorio H, Fournier M, Brice A, Corti O (2006). Biochemical analysis of Parkinson’s disease-causing variants of Parkin, an E3 ubiquitin-protein ligase with monoubiquitylation capacity. Hum Mol Genet.

[162] Jensen LD, Vinther-Jensen T, Kahns S, Sundbye S, Jensen PH (2006). Cellular parkin mutants are soluble under non-stress conditions. NeuroReport.

[163] Clausen L (2024). A mutational atlas for Parkin proteostasis. Nat Commun.

[164] Muller HJ (1932) Further studies on the nature and causes of gene mutations. In: Proceedings of the 6th International Congress of genetics, pp 213–255

[165] Okiyoneda T (2013). Mechanism-based corrector combination restores ΔF508-CFTR folding and function. Nat Chem Biol.

[166] Brusa I (2022). Proteostasis regulators in cystic fibrosis: current development and future perspectives. J Med Chem.

[167] Hutt DM (2010). Reduced histone deacetylase 7 activity restores function to misfolded CFTR in cystic fibrosis. Nat Chem Biol.

[168] Arlow T, Scott K, Wagenseller A, Gammie A (2013). Proteasome inhibition rescues clinically significant unstable variants of the mismatch repair protein Msh2. Proc Natl Acad Sci U S A.

[169] Kampmeyer C (2017). Blocking protein quality control to counter hereditary cancers. Genes Chromosomes Cancer.

[170] Yin Y, Moult J (2019). Characterizing and comparing missense variants in monogenic disease and in cancer. bioRxiv.

[171] Stein A, Fowler DM, Hartmann-Petersen R, Lindorff-Larsen K (2019). Biophysical and mechanistic models for disease-causing protein variants. Trends Biochem Sci.

[172] Clausen L (2019). Protein stability and degradation in health and disease. Adv Protein Chem Struct Biol.

[173] Bartlett AI, Radford SE (2009). An expanding arsenal of experimental methods yields an explosion of insights into protein folding mechanisms. Nat Struct Mol Biol.

[174] Kim YE, Hipp MS, Bracher A, Hayer-Hartl M, Ulrich Hartl F (2013). Molecular chaperone functions in protein folding and proteostasis. Annu Rev Biochem.

[175] Maxwell KL (2005). Protein folding: defining a ‘standard’ set of experimental conditions and a preliminary kinetic data set of two-state proteins. Protein Sci.

[176] Kovalchuke L, Mosharov EV, Levy OA, Greene LA (2019). Stress-induced phospho-ubiquitin formation causes parkin degradation. Sci Rep.

[177] Park HM (2009). The serine protease HtrA2/Omi cleaves Parkin and irreversibly inactivates its E3 ubiquitin ligase activity. Biochem Biophys Res Commun.

[178] Sonia Angeline M, Chaterjee P, Anand K, Ambasta RK, Kumar P (2012). Rotenone-induced parkinsonism elicits behavioral impairments and differential expression of parkin, heat shock proteins and caspases in the rat. Neuroscience.

[179] Aimé P (2015). Trib3 Is Elevated in Parkinson’s disease and mediates death in Parkinson’s disease models. J Neurosci.

[180] Sun X (2013). ATF4 protects against neuronal death in cellular Parkinson’s disease models by maintaining levels of parkin. J Neurosci.

[181] Rakovic A (2013). Phosphatase and tensin homolog (PTEN)-induced putative kinase 1 (PINK1)-dependent ubiquitination of endogenous Parkin attenuates mitophagy: study in human primary fibroblasts and induced pluripotent stem cell-derived neurons. J Biol Chem.

[182] Soleimanpour SA (2014). The diabetes susceptibility gene Clec16a regulates mitophagy. Cell.

[183] Potting C (2018). Genome-wide CRISPR screen for PARKIN regulators reveals transcriptional repression as a determinant of mitophagy. Proc Natl Acad Sci USA.

[184] McLelland GL, Soubannier V, Chen CX, McBride HM, Fon EA (2014). Parkin and PINK1 function in a vesicular trafficking pathway regulating mitochondrial quality control. EMBO J.

[185] Anfinsen CB, Haber E, Sela M, White FH (1961). The kinetics of formation of native ribonuclease during oxidation of the reduced polypeptide chain. Proc Natl Acad Sci U S A.

[186] Hartl FU, Bracher A, Hayer-Hartl M (2011). Molecular chaperones in protein folding and proteostasis. Nature.

[187] Hartl FU, Hayer-Hartl M (2009). Converging concepts of protein folding in vitro and in vivo. Nat Struct Mol Biol.

[188] Rosenzweig R, Nillegoda NB, Mayer MP, Bukau B (2019). The Hsp70 chaperone network. Nat Rev Mol Cell Biol.

[189] Hickey CM, Breckel C, Zhang M, Theune WC, Hochstrasser M (2021). Protein quality control degron-containing substrates are differentially targeted in the cytoplasm and nucleus by ubiquitin ligases. Genetics.

[190] Enam C, Geffen Y, Ravid T, Gardner RG (2018). Protein quality control degradation in the nucleus. Annu Rev Biochem.

[191] Abildgaard AB (2020). Co-Chaperones in targeting and delivery of misfolded proteins to the 26S proteasome. Biomolecules.

[192] Alberti S, Esser C, Höhfeld J (2003). BAG-1–a nucleotide exchange factor of Hsc70 with multiple cellular functions. Cell Stress Chaperones.

[193] Andersen KM, Hofmann K, Hartmann-Petersen R (2005). Ubiquitin-binding proteins: similar, but different. Essays Biochem.

[194] Rose JM, Novoselov SS, Robinson PA, Cheetham ME (2011). Molecular chaperone-mediated rescue of mitophagy by a Parkin RING1 domain mutant. Hum Mol Genet.

[195] Schlehe JS (2008). Aberrant folding of pathogenic Parkin mutants: aggregation versus degradation. J Biol Chem.

[196] Zhang CW (2016). Pharmacological or genetic activation of Hsp70 protects against loss of parkin function. Neurodegener Dis.

[197] Winklhofer KF, Henn IH, Kay-Jackson PC, Heller U, Tatzelt J (2003). Inactivation of parkin by oxidative stress and C-terminal truncations: a protective role of molecular chaperones. J Biol Chem.

[198] Kalia SK (2004). BAG5 inhibits parkin and enhances dopaminergic neuron degeneration. Neuron.

[199] Hu S (2021). Molecular chaperones and Parkinson’s disease. Neurobiol Dis.

[200] Kalia KS, Kalia LV, McLean JP (2010). Molecular chaperones as rational drug targets for Parkinson’s disease therapeutics. CNS Neurol Disord Drug Targets.

[201] Kampmeyer C (2022). Disease-linked mutations cause exposure of a protein quality control degron. Structure.

[202] Timms RT, Koren I (2020). Tying up loose ends: the N-degron and C-degron pathways of protein degradation. Biochem Soc Trans.

[203] Johansson KE, Mashahreh B, Hartmann-Petersen R, Ravid T, Lindorff-Larsen K (2023). Prediction of quality-control degradation signals in yeast proteins. J Mol Biol.

[204] Mashahreh B (2022). Conserved degronome features governing quality control associated proteolysis. Nat Commun.

[205] Koren I (2018). The eukaryotic proteome is shaped by E3 ubiquitin ligases targeting C-terminal degrons. Cell.

[206] Abildgaard AB (2023). HSP70-binding motifs function as protein quality control degrons. Cell Mol Life Sci.

[207] Maurer MJ (2016). Degradation signals for ubiquitin-proteasome dependent cytosolic protein quality control (CytoQC) in yeast. G3 (Bethesda).

[208] Zhang Z (2023). Elucidation of E3 ubiquitin ligase specificity through proteome-wide internal degron mapping. Mol Cell.

[209] Ravid T, Hochstrasser M (2008). Diversity of degradation signals in the ubiquitin-proteasome system. Nat Rev Mol Cell Biol.

[210] Stevens MU (2023). Structure-based design and characterization of Parkin-activating mutations. Life Sci Alliance.

[211] Van Hout CV (2020). Exome sequencing and characterization of 49,960 individuals in the UK Biobank. Nature.

[212] Starita LM (2017). Variant interpretation: functional assays to the rescue. Am J Human Genet.

[213] Weile J, Roth FP (2018). Multiplexed assays of variant effects contribute to a growing genotype-phenotype atlas. Hum Genet.

[214] Fowler DM (2023). An Atlas of Variant Effects to understand the genome at nucleotide resolution. Genome Biol.

[215] Cheng J et al (2023) Accurate proteome-wide missense variant effect prediction with AlphaMissense. Science 381:eadg7492 10.1126/science.adg7492 37733863

[216] Frazer J (2021). Disease variant prediction with deep generative models of evolutionary data. Nature.

[217] Tabet D, Parikh V, Mali P, Roth FP, Claussnitzer M (2022). Scalable functional assays for the interpretation of human genetic variation. Annu Rev Genet.

[218] Shlevkov E (2022). Discovery of small-molecule positive allosteric modulators of Parkin E3 ligase. iScience.

[219] Chiti F, Kelly JW (2022). Small molecule protein binding to correct cellular folding or stabilize the native state against misfolding and aggregation. Curr Opin Struct Biol.

[220] Wang F (2022). USP30: structure, emerging physiological role, and target inhibition. Front Pharmacol.

